# Demographics and clinical characteristics of Indian patients with heart failure visiting outpatient clinics: a cross-sectional observational study

**DOI:** 10.1093/eschf/xvag236

**Published:** 2026-09-10

**Authors:** Kamal Sharma, J C Mohan, Shantanu Sengupta, Saumitra Ray, Ajit Mullasari, Johann Christopher, P B Jayagopal, Hiren Prajapati, Willem J Verberk

**Affiliations:** SAL Hospital, Ahmedabad, Gujarat, India; Institute of Heart & Vascular Diseases, Jaipur Golden Hospital, Delhi, India; Sengupta Hospital and Research Institute, Nagpur, India; Department of Cardiology, Manipal Hospitals, Kolkata, West Bengal, India; Department of Cardiology, Madras Medical Mission, Chennai, India; Department of Cardiac Imaging, CARE Hospitals, Hyderabad, India; Lakshmi Hospital, Palakkad, Kerala, India; Medical Affairs, Eris Lifesciences Limited, Ahmedabad, Gujarat, India; CARIM School for Cardiovascular Diseases, Maastricht University, Maastricht, The Netherlands; Mexlid, Research in Life Sciences and Medical Devices, Gennep, The Netherlands

**Keywords:** Heart failure, Ejection fraction, India, HFpEF, HFrEF, Observational study

## Abstract

**Background:**

Data on heart failure (HF) phenotypes in India remain limited. We examined clinical characteristics and factors associated with HF phenotype defined according to ejection fraction (EF) in a large multicentre outpatient cohort.

**Methods:**

We performed a retrospective cross-sectional analysis of adult HF outpatients with documented EF at centres across India (April 2021–March 2023). Patients were classified as HFrEF (EF ≤40%), HFmrEF (41%–49%), or HFpEF (≥50%); multivariable logistic regression identified factors associated with phenotype.

**Results:**

Among 1207 patients (62 ± 10 years; 73% male), HFrEF was present in 822 (68.1%), HFmrEF in 89 (7.4%), and HFpEF in 296 (24.5%). Patients with HFpEF had higher systolic blood pressure (153 ± 26 vs 122 ± 25 mmHg), higher body mass index (BMI; 27.5 ± 4.9 vs 26.4 ± 4.4 kg/m^2^), and more chronic kidney disease (45% vs 23%) and stroke (27% vs 6.2%) than those with HFrEF (all *P* ≤ .003). Ischaemic heart disease (66% vs 56%; *P* = .002) and higher NT-proBNP [median 3705 (481–6232) vs 140 (121–521) pg/ml; *P* < .001) were more common in HFrEF. In multivariable analysis, higher BMI [odds ratio (OR) 1.35 per 5 kg/m^2^, 95% confidence interval (CI) 1.10–1.65], higher systolic blood pressure (OR 1.48 per 10 mmHg, 95% CI 1.37–1.60), and stroke (OR 1.99, 95% CI 1.11–3.54) were associated with higher odds of HFpEF, whereas higher NT-proBNP (OR 0.46, 95% CI 0.40–0.53) and diabetes (OR 0.56, 95% CI 0.38–0.83) were associated with lower odds of HFpEF.

**Conclusions:**

Higher BMI and blood pressure were associated with HFpEF, whereas elevated NT-proBNP and diabetes were associated with HFrEF. These cross-sectional associations may support phenotype-specific management of HF.

## Graphical Abstract

**Graphical Abstract:**
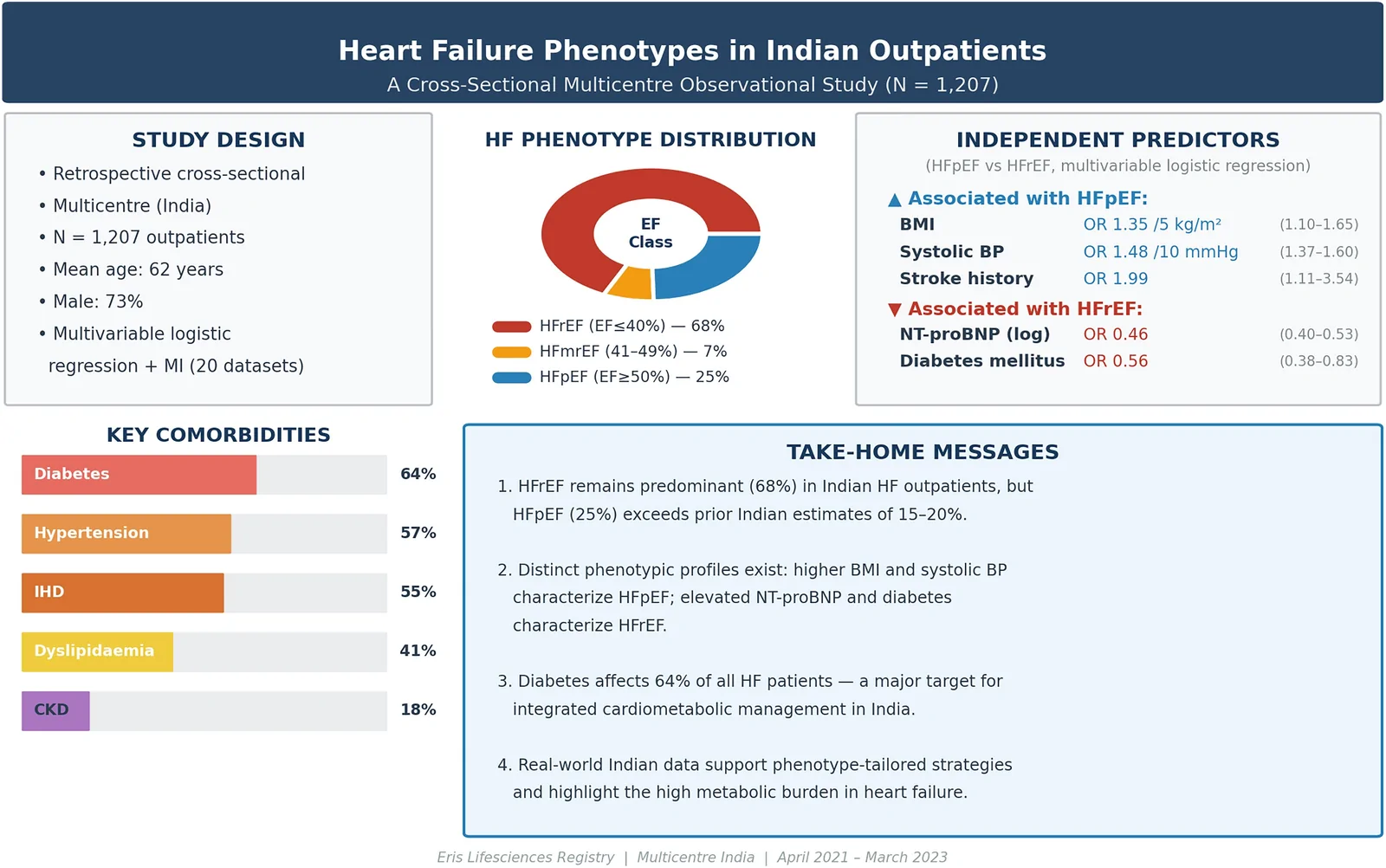
Graphical abstract summarizing a retrospective cross-sectional multicentre study of 1,207 Indian heart failure outpatients. HFrEF was the predominant phenotype at 68%, followed by HFpEF at 25% and HFmrEF at 7%. Diabetes, hypertension, ischaemic heart disease, dyslipidaemia and chronic kidney disease were common comorbidities. Higher BMI and systolic blood pressure were independently associated with HFpEF, whereas higher NT-proBNP and diabetes were associated with HFrEF. The findings highlight distinct heart failure phenotypes and a high cardiometabolic burden among Indian outpatients.

## Introduction

Heart failure is a chronic and progressive clinical syndrome and a major global health problem.^1^ In India, the estimated prevalence ranges from 1.3 to 4.6 million patients, causing a large burden for a low- and middle-income country.^2^ The epidemiology of heart failure in India differs from that reported in high-income countries. Patients in India often present at a younger age, and rheumatic heart disease and different comorbidity patterns remain more common.^3^

Heart failure with reduced ejection fraction (HFrEF) has always been considered the most common form of heart failure.^4^ However, recent studies suggest that the proportion of patients with heart failure with preserved ejection fraction (HFpEF) is increasing.^5^ In community-based studies from high-income countries, approximately half of patients with heart failure have HFpEF, and this proportion continues to rise.^1,6^ Data from India are predominantly hospital-based: in the National Heart Failure Registry, which enrolled patients admitted with acute heart failure across 53 centres, 12.7% had preserved EF.^3^

The evaluation of heart failure is based on clinical assessment together with laboratory and imaging investigations.^7^ Routine laboratory tests include complete blood count, urinalysis, and metabolic profile. Heart failure-specific investigations include natriuretic peptides, chest radiography, and transthoracic echocardiography.^8,9^ These diagnostic approaches are in line with recommendations from the American College of Cardiology and the European Society of Cardiology.^10,11^ Common comorbidities, such as hypertension, diabetes mellitus, coronary artery disease, and chronic kidney disease, are associated with worse clinical outcomes.^12^ HFpEF and HFrEF are recognized as distinct phenotypes with important differences in pathophysiology, comorbidities, and treatment response.^13–16^

Despite increasing recognition of the heterogeneity of heart failure, recent data from Indian outpatient populations remain limited. Most published registries from India have focused on hospitalized patients or those with acute decompensated heart failure.^3,17^ In addition, factors associated with variation in ejection fraction across the spectrum of heart failure have not been systematically studied in Indian populations.

This cross-sectional observational study aimed to describe the distribution of heart failure phenotypes and the clinical characteristics of patients attending outpatient departments across India. The study also aimed to identify independent factors associated with heart failure phenotype using multivariable regression modelling.

## Methods

### Study design and participants

This retrospective, cross-sectional, observational multicentre study was conducted across India. The study included medical records of patients who attended the participating outpatient cardiology clinics between April 2021 and March 2023. Data were retrospectively abstracted between June 2023 and April 2024. The study protocol (ERIS/OS/23/015) was approved by the Medilink Ethics Committee in June 2023. The study was conducted in accordance with the Declaration of Helsinki and the ICMR National Ethical Guidelines for Biomedical and Health Research involving Human Participants. Written informed consent was obtained from all participants.

This study is reported in accordance with the Strengthening the Reporting of Observational Studies in Epidemiology (STROBE) guidelines. As the analysis was based on data extracted from routine clinical records, the study was conducted in line with the CODE-EHR minimum standards for the use of structured healthcare data in research.

Data were retrospectively abstracted from the medical records of the participating outpatient clinics into a structured electronic case report form with prespecified fields. Variables were defined by the case report form, and no administrative diagnostic coding system was used. Comorbidities, potential causes of heart failure, and medication use were recorded as prespecified fields. Data quality checks were applied using prespecified plausibility ranges, with values outside these ranges treated as missing; unrecorded or unobtainable data were likewise treated as missing.

In accordance with the Sex and Gender Equity in Research (SAGER) guidelines, both sexes were included without restriction. Sex was recorded as documented in the medical records and was included as a covariate in the regression models. Results are reported stratified by sex where relevant.

### Patients

Adults aged 18 years or older with a diagnosis of heart failure and a documented quantitative ejection fraction (EF) measurement were included. Heart failure phenotype was classified according to EF as HFrEF (EF ≤40%), HF with mildly reduced EF (HFmrEF; EF 41%–49%), or HFpEF (EF ≥50%).^10,11^ In line with the 2021 ESC and 2022 AHA/ACC/HFSA universal definitions, an EF of exactly 40% was classified as HFrEF.^18^

Among 1250 patients with a documented quantitative EF, 43 had values outside the prespecified plausible range and were excluded, leaving 1207 patients in the EF-phenotyping analytical cohort.

All included patients had a diagnosis of heart failure established by the treating cardiologist in routine clinical practice; for the present analysis, they were classified into HF phenotypes according to the guideline-based EF thresholds given above. Because this was a retrospective analysis of patients with an established clinical diagnosis of heart failure, additional echocardiographic parameters (such as the E/e′ ratio or left atrial volume index) or natriuretic peptide thresholds were not required for study phenotyping. NT-proBNP concentrations were available in 810 of 1207 patients overall (67.1%), including 263 of 296 patients classified as HFpEF (88.9%). Patients with an alternative primary explanation for symptoms or volume overload, such as chronic kidney disease, were not excluded, and specific HFpEF aetiologies such as hypertrophic cardiomyopathy or cardiac amyloidosis were neither systematically excluded nor captured in the case report form.

Data extracted from medical records included demographic characteristics, anthropometric measurements, lifestyle factors, vital signs, comorbidities, medication use, NT-proBNP concentrations, and echocardiographic findings.

Comorbidities included hypertension, diabetes mellitus, coronary artery disease, chronic kidney disease, stroke, and dyslipidaemia. Potential causes of heart failure were recorded, including ischaemic heart disease, hypertensive heart disease, dilated cardiomyopathy, heart failure following acute coronary syndrome, and rheumatic heart disease. Patients could have more than one recorded cause of heart failure.

### Statistical analysis

Continuous variables are presented as mean (standard deviation) for normally distributed data and median (interquartile range) for skewed data. Categorical variables are presented as counts and percentages. Missing values for each variable are reported in *Table 1*.

**Table 1:** Baseline demographic, clinical, and echocardiographic characteristics of patients according to heart failure phenotype

	Heart failure phenotype				
Characteristic	Overall *N* = 1 207^a^	HFrEF *N* = 822^a^	HFmrEF *N* = 89^a^	HFpEF *N* = 296^a^	*P*-value^b^
Age (years)	62 (10)	61 (9)	62 (11)	62 (11)	.724
Sex					.109
Female	326 (27%)	234 (28%)	26 (29%)	66 (22%)	
Male	881 (73%)	588 (72%)	63 (71%)	230 (78%)	
Height (cm)	163 (10)	163 (10)	163 (9)	164 (10)	.221
Weight (kg)	71 (11)	70 (11)	73 (16)	73 (10)	<.001
BMI (kg/m^2^)	26.8 (4.6)	26.4 (4.4)	28.1 (4.9)	27.5 (4.9)	.003
**BMI ≥23 kg/m^2^**	999 (83%)	664 (81%)	73 (84%)	262 (89%)	.011
**BMI ≥25 kg/m^2^**	781 (65%)	520 (63%)	60 (69%)	201 (68%)	.261
**BMI ≥27.5 kg/m^2^**	496 (41%)	322 (39%)	50 (57%)	124 (42%)	.004
**BMI ≥30 kg/m^2^**	263 (22%)	161 (20%)	31 (36%)	71 (24%)	.002
**BMI ≥40 kg/m^2^**	16 (1.3%)	6 (0.7%)	1 (1.1%)	9 (3.0%)	.012
Systolic blood pressure (mmHg)	130 (28)	122 (25)	126 (22)	153 (26)	<.001
Diastolic blood pressure (mmHg)	85 (16)	80 (11)	80 (12)	100 (20)	<.001
Heart rate (bpm)	85 (14)	85 (14)	81 (12)	87 (15)	<.001
NT-proBNP (pg/mL), median (IQR)	1000 (140, 4600)	3705 (481, 6232)	700 (130, 2600)	140 (121, 521)	<.001
Missing, *n*	397	318	46	33	
Hypertension	1024 (85%)	686 (83%)	69 (78%)	269 (91%)	.001
Coronary artery disease	569 (47%)	382 (46%)	40 (45%)	147 (50%)	.584
Diabetes mellitus	768 (64%)	535 (65%)	55 (62%)	178 (60%)	.295
Chronic kidney disease	330 (27%)	187 (23%)	11 (12%)	132 (45%)	<.001
Stroke	133 (11%)	51 (6.2%)	3 (3.4%)	79 (27%)	<.001
Dyslipidaemia	757 (63%)	506 (62%)	57 (64%)	194 (66%)	.461
**Rhythm disturbances**	76 (6.3%)	48 (5.8%)	7 (7.9%)	21 (7.1%)	.612
Ischaemic heart conditions	761 (63%)	546 (66%)	48 (54%)	167 (56%)	.002
Hypertensive heart disease	408 (34%)	240 (29%)	25 (28%)	143 (48%)	<.001
**Dilated cardiomyopathy**	269 (22%)	162 (20%)	24 (27%)	83 (28%)	.007
Rheumatic heart disease	119 (9.9%)	60 (7.3%)	6 (6.7%)	53 (18%)	<.001
HF after acute coronary syndrome	315 (26%)	211 (26%)	16 (18%)	88 (30%)	.076

Continuous variables are presented as mean ± standard deviation or median (interquartile range), and categorical variables as number (%). *P* values were calculated using Kruskal-Wallis tests for continuous variables and χ2 or Fisher exact tests for categorical variables.

^a^Mean (SD); *n* (%); Median (Q1, Q3)

^b^Kruskal–Wallis rank sum test; Pearson's Chi-squared test; Fisher's exact test

Between-group comparisons were performed using the Kruskal–Wallis test for continuous variables and the χ2 test or Fisher exact test for categorical variables, as appropriate.

The primary multivariable analysis used logistic regression to identify factors associated with HFpEF (EF ≥50%) compared with HFrEF (EF ≤40%). Patients with HFmrEF were excluded from this binary comparison. Predictors were selected *a priori* based on clinical relevance and included age, sex, body mass index, systolic blood pressure, ln(1 + NT-proBNP), diabetes mellitus, chronic kidney disease, stroke, dyslipidaemia, ischaemic heart disease, and rheumatic heart disease. Descriptive analyses and *Table 1* were based on observed data, with missingness reported for each variable. Missing predictor values in the multivariable logistic regression were handled using multiple imputation with 20 imputed datasets. Results were pooled using Rubin’s rules and are presented as odds ratios with 95% confidence intervals.

BMI was additionally categorized using thresholds relevant to Asian populations (≥23, ≥25, and ≥27.5 kg/m^2^), as well as BMI-defined obesity (≥30 kg/m^2^) and severe obesity (≥40 kg/m^2^). Patients included in the analytical cohort were descriptively compared with patients excluded because their recorded quantitative EF was outside the prespecified plausible range using absolute standardized mean differences.

All analyses were performed using R version 4.3.3 (R Foundation for Statistical Computing, Vienna, Austria). A two-sided *P* value < .05 was considered statistically significant.

## Results

### Patient characteristics

Of 1250 patients with a documented quantitative EF, 43 had values outside the prespecified plausible range and were excluded, leaving 1207 patients in the EF-phenotyping analytical cohort (Supplementary Figure S1). The 43 excluded patients were similar to the analytical cohort in age, sex, height and heart rate (absolute standardized mean differences ≤0.15) but had higher recorded weight, body mass index and blood pressure (absolute standardized mean differences 0.50–0.96) (Supplementary Table S1).

*Table 1*. Patient characteristics by heart failure phenotype

A total of 1207 outpatients with documented quantitative EF were analysed. HFrEF was the most common phenotype (822, 68.1%), followed by HFpEF (296, 24.5%) and HFmrEF (89, 7.4%).

Baseline characteristics differed across phenotypes (*Table 1*). Compared with HFrEF, patients with HFpEF had higher systolic blood pressure (153 ± 26 vs 122 ± 25 mmHg, *P* < .001), higher diastolic blood pressure (100 ± 20 vs 80 ± 11 mmHg, *P* < .001), and higher BMI (27.5 ± 4.9 vs 26.4 ± 4.4 kg/m^2^, *P* = .003). Chronic kidney disease (45% vs 23%, *P* < .001), stroke (27% vs 6.2%, *P* < .001), hypertensive heart disease (48% vs 29%, *P* < .001), and rheumatic heart disease (18% vs 7.3%, *P* < .001) were more common in HFpEF than HFrEF. In contrast, ischaemic heart disease was more common in HFrEF than HFpEF (66% vs 56%, *P* = .002). Median NT-proBNP was highest in HFrEF and lowest in HFpEF [3705 (481, 6232) vs 140 (121, 521) pg/ml, *P* < .001). Using BMI categories relevant to Asian populations, BMI was ≥23 kg/m^2^ in 999 of the 1204 patients with a recorded BMI (83.0%), ≥25 kg/m^2^ in 781 (64.9%), and ≥27.5 kg/m^2^ in 496 (41.2%), with differences across phenotypes at the ≥23 and ≥27.5 kg/m^2^ thresholds (*P* = .011 and *P* = .004) but not at ≥25 kg/m^2^ (*P* = .261) (*Table 1*).

Rhythm disturbances were recorded as a comorbidity in 5.8% of patients with HFrEF, 7.9% with HFmrEF, and 7.1% with HFpEF (*P* = .612), and dilated cardiomyopathy was recorded as a potential cause of heart failure in 19.7%, 27.0%, and 28.0%, respectively (*P* = .007) (*Table 1*).

### Cardiometabolic profile of patients with HFpEF

Among patients with HFpEF, arterial hypertension was present in 269 of 296 patients (90.9%), diabetes mellitus in 178 (60.1%), dyslipidaemia in 194 (65.5%), and chronic kidney disease in 132 (44.6%). A BMI ≥27.5 kg/m^2^ was present in 124 patients (41.9%), BMI-defined obesity (BMI ≥30 kg/m^2^) in 71 (24.0%), and severe obesity (BMI ≥40 kg/m^2^) in 9 (3.0%) (*Table 1*).

### Recorded causes of heart failure in HFrEF

Among the 822 patients with HFrEF, the major recorded causes of heart failure were as follows. Ischaemic heart conditions were recorded in 546 (66.4%) and heart failure following an acute coronary syndrome in 211 (25.7%); taken together, 642 patients (78.1%) had an ischaemic cause recorded. Dilated cardiomyopathy was recorded in 162 patients (19.7%), of whom 73 (8.9% of all HFrEF) had no concomitant ischaemic cause, consistent with non-ischaemic dilated cardiomyopathy. Hypertensive heart disease was recorded in 240 (29.2%) and rheumatic heart disease in 60 (7.3%).

### Medication use by heart failure phenotype

*Table 2*. Medication use by heart failure phenotype.

**Table 2:** Medication use according to heart failure phenotype

	Heart failure phenotype				
Medication	Overall *N* = 1 207^a^	HFrEF *N* = 822^a^	HFmrEF *N* = 89^a^	HFpEF *N* = 296^a^	*P*-value^b^
ARNI	889 (74%)	584 (71%)	60 (67%)	245 (83%)	<.001
SGLT2 inhibitor	890 (74%)	657 (80%)	52 (58%)	181 (61%)	<.001
Digoxin	239 (20%)	130 (16%)	16 (18%)	93 (31%)	<.001
Beta-blocker	928 (77%)	643 (78%)	69 (78%)	216 (73%)	.183
ACE inhibitor	501 (42%)	315 (38%)	26 (29%)	160 (54%)	<.001
ARB	466 (39%)	344 (42%)	17 (19%)	105 (35%)	<.001
MRA	329 (27%)	272 (33%)	19 (21%)	38 (13%)	<.001
Vericiguat	80 (6.6%)	54 (6.6%)	1 (1.1%)	25 (8.4%)	.051
Diuretic	618 (51%)	479 (58%)	49 (55%)	90 (30%)	<.001

Data are presented as number (%). *P* values were calculated using χ2 tests.

^a^ *n* (%)

^b^Pearson's χ^2^ test.

Medication use differed by heart failure phenotype (*Table 2*). ARNI use was common overall (74%) and highest in HFpEF (83%), compared with HFrEF (71%) and HFmrEF (67%; *P* < .001). SGLT2 inhibitor use was highest in HFrEF (80%) and lower in HFmrEF (58%) and HFpEF (61%; *P* < .001). MRA and diuretic use were also higher in HFrEF than in HFpEF (33% vs 13% and 58% vs 30%, respectively; both *P* < .001). Digoxin was used in 31% of patients with HFpEF compared with 16% with HFrEF (*P* < .001). Vericiguat was used in 8.4% of patients with HFpEF, 6.6% with HFrEF and 1.1% with HFmrEF (*P* = .051).

### Ejection fraction distribution by sex

*Figure 1* shows the distribution of EF by sex. Most patients had EF values in the reduced range. Male patients accounted for the majority of patients across the EF spectrum, in line with their greater representation in the cohort. Women represented 27% of the study population and were also predominantly observed in the reduced EF range.

**Figure 1:**
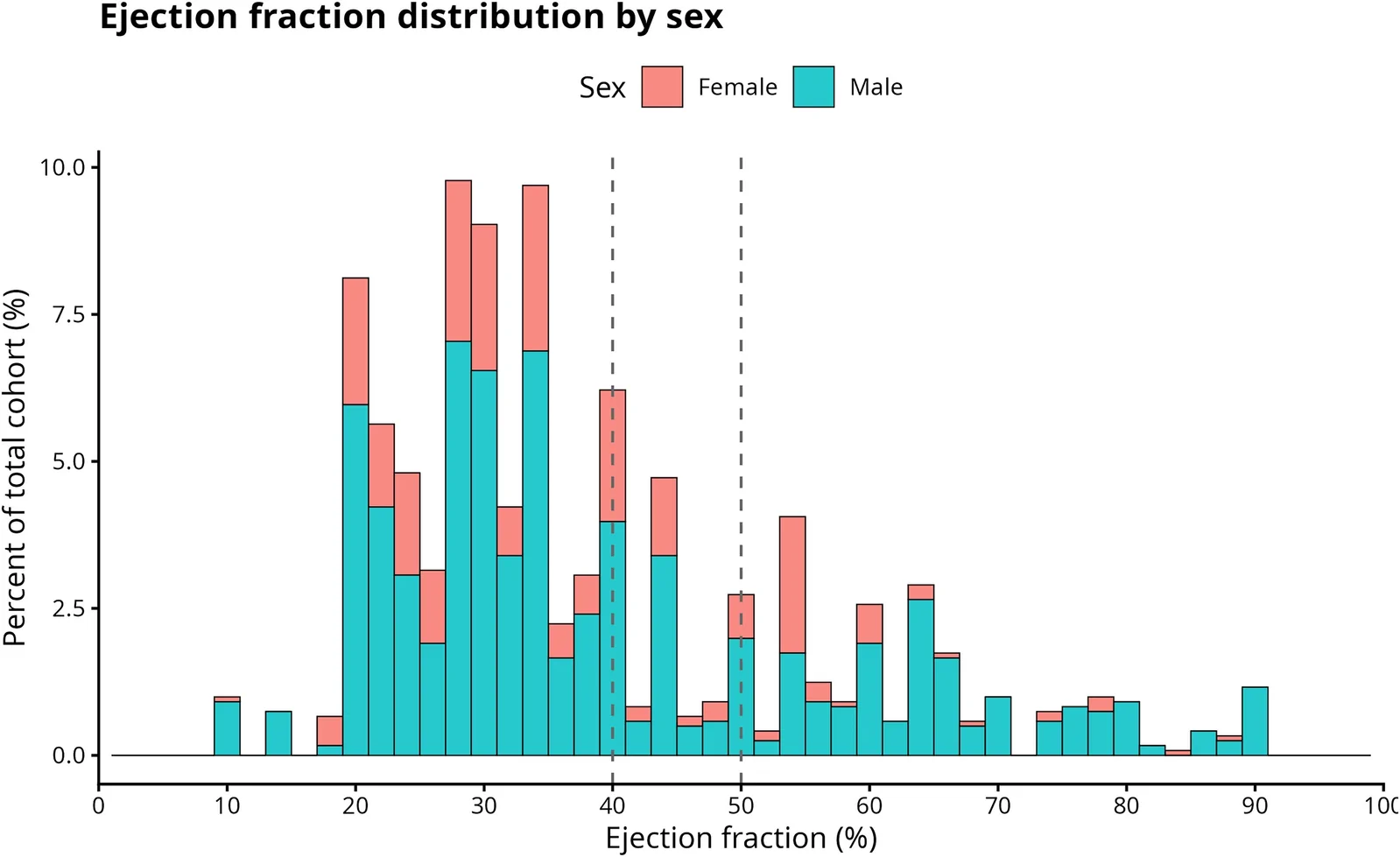
Distribution of ejection fraction according to sex. Histogram showing the distribution of ejection fraction in male and female patients. Vertical dashed lines indicate EF thresholds used to define HFrEF (≤40%), HFmrEF (41%–49%), and HFpEF (≥50%)

Formal comparison showed that women had a lower mean EF than men (36.9% vs 39.9%; *P* = .003). However, the distribution of heart failure phenotypes (HFrEF, HFmrEF, and HFpEF) did not differ significantly between women and men (*P* = .109).

### Logistic regression model

Compared with HFrEF, HFpEF was independently associated with stroke [odds ratio (OR) 1.99, 95% CI 1.11–3.54], systolic blood pressure (OR 1.48, 95% CI 1.37–1.60) and higher BMI (OR 1.35, 95% CI 1.10–1.65). Higher NT-proBNP levels (OR 0.46, 95% CI 0.40–0.53) and diabetes (OR 0.56, 95% CI 0.38–0.83) were associated with lower odds of HFpEF. No significant associations were observed for age, sex, chronic kidney disease, dyslipidaemia, ischaemic heart disease, or rheumatic heart disease (*Figure 2*).

**Figure 2:**
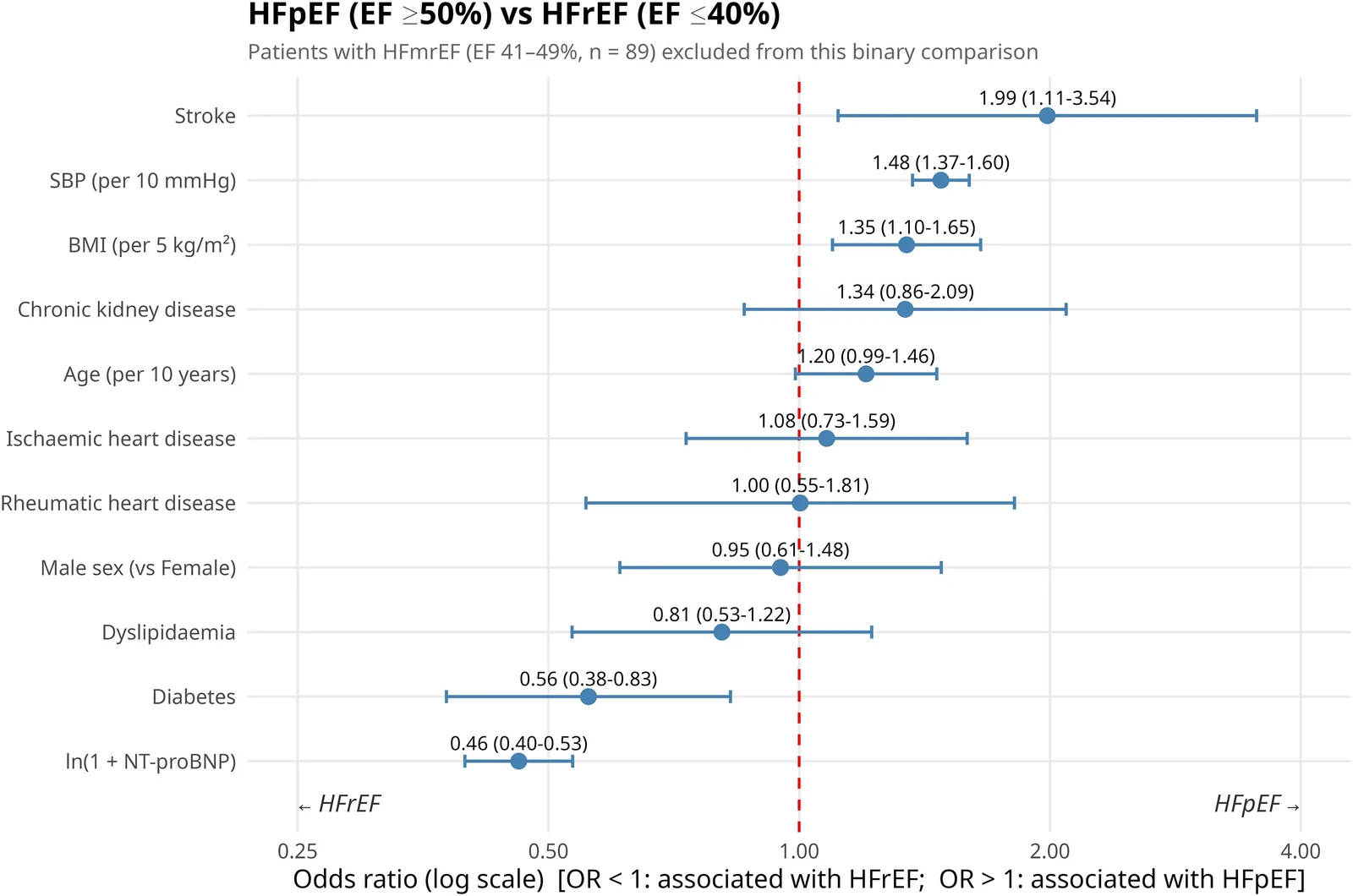
Forest plot of pooled odds ratios for HFpEF (EF ≥50%) vs HFrEF (EF ≤40%) from multivariable logistic regression analysis (*n* = 1118; 822 HFrEF + 296 HFpEF). Patients with HFmrEF (EF 41%–49%, *n* = 89) were excluded from this binary analysis. Odds ratios are shown with 95% confidence intervals, pooled across 20 imputed datasets using Rubin’s rules. OR > 1 indicates greater odds of HFpEF; OR < 1 indicates greater odds of HFrEF

## Discussion

### Principal findings

In this study of outpatients with heart failure across India, EF phenotypes showed clear clinical differences. Patients with HFpEF had higher blood pressure, higher BMI, and a higher prevalence of chronic kidney disease and stroke, whereas patients with HFrEF more commonly had ischaemic heart disease and higher NT-proBNP concentrations. In multivariable analyses, higher BMI, systolic blood pressure, and history of stroke were independently associated with higher odds of HFpEF, while higher NT-proBNP concentrations and diabetes were associated with lower odds of HFpEF.

### Phenotype distribution and clinical characteristics

The phenotype distribution observed in the present study was comparable with recent European registry data, in which HFrEF accounted for 57% of cases, HFmrEF for 17%, and HFpEF for 26%.^19^ The proportion of HFpEF in our outpatient cohort (24.5%) was higher than reported in Indian registries of hospitalized acute heart failure, in which HFpEF accounted for approximately 13%–14% of admissions^3,20^; this difference may partly reflect the outpatient setting of the present study.

Patients with HFpEF had a higher BMI and a higher prevalence of hypertension and stroke. These findings are consistent with the model proposed by Paulus and Tschöpe, which describes HFpEF as a condition driven by multiple comorbidities, particularly metabolic disease and hypertension, leading to inflammation, small vessel dysfunction, and structural changes in the heart.^21^

Stroke was independently associated with HFpEF and should be interpreted as part of a broader vascular comorbidity profile, not as evidence of causality. In this cohort, stroke clustered with higher blood pressure, higher BMI, chronic kidney disease, and hypertensive heart disease. This finding supports careful assessment of cerebrovascular history, blood pressure control, rhythm disorders, and secondary prevention in outpatient HFpEF care.

Although chronic kidney disease was substantially more common in HFpEF than in HFrEF (45% vs 23%), this association was no longer observed in the multivariable model. This suggests that the observed difference was largely explained by the clustering of chronic kidney disease with other cardiometabolic characteristics associated with HFpEF, rather than by an independent association between chronic kidney disease and heart failure phenotype.

HFmrEF did not show a clearly distinct clinical profile and appeared to share characteristics with both neighbouring phenotypes. This supports the concept of HFmrEF as an intermediate or transitional phenotype.^22^

In the current cohort, diabetes was highly prevalent, affecting 64% of the study population. This high prevalence may be explained by the substantial burden of diabetes commonly reported in heart failure registries,^23^ as well as the high background prevalence of type 2 diabetes in the Indian population.^24^ Although the prevalence of diabetes did not differ significantly between the study groups, diabetes was independently associated with HFrEF in the multivariable analysis. This association is biologically plausible, as diabetes contributes to myocardial fibrosis, adverse ventricular remodelling, impaired cardiac energy metabolism, and accelerated coronary artery disease, all of which may promote the development and progression of HFrEF.^25,26^

NT-proBNP concentrations showed a clear gradient across heart failure phenotypes, with the highest values observed in HFrEF and the lowest in HFpEF. This finding is consistent with previous studies.^12,27^ Higher NT-proBNP concentrations in HFrEF likely reflect greater myocardial wall stress, volume overload, and more severe systolic dysfunction, resulting in increased synthesis and release of natriuretic peptides by the myocardium.^28^

### Sex-related observations

In many Western cohorts, women are more commonly represented in HFpEF and men in HFrEF.^29^ In the present cohort, however, women were more often observed in the lower EF range, although the distribution of HF phenotypes did not differ significantly between women and men. The reasons for this pattern cannot be determined from the present cross-sectional data.

### Therapeutic patterns across heart failure phenotypes

ARNI and SGLT2 inhibitors were the most frequently prescribed therapies in the cohort, with each used in 74% of patients overall. SGLT2 inhibitor use was highest in HFrEF (80%) and lower in HFmrEF (58%) and HFpEF (61%), consistent with recent registry data and the earlier establishment of SGLT2 inhibitors as disease-modifying therapy in HFrEF.^30,31^

In contrast, ARNI use was highest in HFpEF (83%), followed by HFrEF (71%) and HFmrEF (67%). This differs from most registries, including the ESC Heart Failure III Registry, where ARNI use is largely concentrated in HFrEF and remains uncommon in HFpEF.^19^ Historical EF measurements, treatment indications, dose, duration, and physician rationale were unavailable. It was therefore not possible to determine whether ARNI treatment in some patients classified as HFpEF had been initiated during a period of lower EF or for another clinical reason, and the high ARNI use in HFpEF should be interpreted cautiously.

MRAs and diuretics were prescribed more frequently in HFrEF than in HFpEF. However, only one-third of patients with HFrEF received an MRA, indicating an opportunity for further treatment optimization given the established role of MRAs in HFrEF.^31^ Digoxin was more frequently prescribed in HFpEF than in HFrEF (31% vs 16%). Because atrial fibrillation was not recorded separately, its contribution to digoxin use could not be determined. Recorded rhythm disturbances and device implantation were similarly uncommon among digoxin users and non-users, and the available data therefore do not explain the relatively high use of digoxin in HFpEF.

Vericiguat was used in 8.4% of patients classified as HFpEF. Evidence supporting vericiguat derives from patients with worsening heart failure and reduced EF in VICTORIA, whereas VITALITY-HFpEF did not demonstrate an improvement in physical limitation in patients with HFpEF.^32,33^ Historical EF measurements, treatment indications, and prescribing rationale were unavailable, and the reason for vericiguat use in these patients could therefore not be established.

Overall, uptake of contemporary heart failure therapies was reasonably high, particularly for ARNI and SGLT2 inhibitors. However, treatment patterns did not always follow the expected phenotype-specific distribution, while MRA use in HFrEF was comparatively low and represents an area for further optimization. Similar gaps in optimal treatment use have been reported in the Associates for Cardiology Education and Research Chronic Heart Failure (ACERT CHEF) registry.^34^

### Contemporary treatment considerations in HFpEF

The HFpEF group was characterized by a high prevalence of hypertension (91%), diabetes (60%), dyslipidaemia (66%), and chronic kidney disease (45%); BMI-defined obesity (BMI ≥30 kg/m^2^) was present in 24%. This cardiometabolic profile is relevant to contemporary management of HFpEF. EMPEROR-Preserved and DELIVER demonstrated reductions in heart failure events with empagliflozin and dapagliflozin, respectively, and SGLT2 inhibitors carry a class I recommendation for HFpEF in the 2023 focused update of the ESC guidelines.^11,35,36^ Because our study covered April 2021 to March 2023, when evidence supporting SGLT2 inhibition in HFpEF was still emerging, the observed treatment patterns should be interpreted in this temporal context.

Evidence for MRAs in HFpEF has evolved. In TOPCAT, spironolactone did not significantly reduce the primary composite outcome, although heart failure hospitalization was reduced.^37^ More recently, the non-steroidal MRA finerenone reduced the composite of total worsening heart failure events and cardiovascular death in patients with HFmrEF or HFpEF in FINEARTS-HF.^38^ Sacubitril–valsartan did not significantly reduce the primary outcome in PARAGON-HF, although possible benefit was observed in selected subgroups.^39^ Management of hypertension, diabetes, kidney disease, congestion, and other relevant comorbidities remains an important component of HFpEF care.^40^

Weight-directed treatment is particularly relevant in HFpEF with obesity. In STEP-HFpEF, semaglutide improved symptoms and physical limitations and produced substantial weight loss in patients with HFpEF and obesity.^41^ In SUMMIT, tirzepatide improved health status and reduced the composite of cardiovascular death or worsening heart failure in patients with HFpEF and obesity.^42^ In our cohort, 24% of patients with HFpEF had BMI-defined obesity using the conventional threshold of ≥30 kg/m^2^, increasing to 42% when the Asian-specific threshold of ≥27.5 kg/m^2^ was applied, identifying a clinically relevant subgroup in whom weight management may be considered alongside other appropriate HFpEF treatment.

### Clinical implications

The findings support a phenotype-based approach to outpatient heart failure management. HFpEF was more strongly associated with hypertension, metabolic comorbidities, and a history of stroke, whereas HFrEF was characterized by higher NT-proBNP concentrations and a greater burden of ischaemic heart disease. The marked differences in NT-proBNP concentrations across HF phenotypes support the routine use of natriuretic peptide testing in outpatient evaluation.

### Strengths and limitations

This study represents one of the larger contemporary analyses of HF phenotypes in Indian outpatient practice. The multicentre design included patients from different outpatient settings across India. To reduce dependence on clinician-recorded HF phenotype, patients were classified using the same quantitative EF thresholds throughout the cohort.

Several limitations should be considered. The cross-sectional design does not allow conclusions about causality, changes in HF phenotype, or longitudinal outcomes. Stroke was recorded only as a baseline comorbidity, and information on stroke subtype, timing, severity, recurrent events, atrial fibrillation, anticoagulation, and secondary prevention was unavailable. The association between stroke and HFpEF should therefore be interpreted as a marker of vascular comorbidity rather than a causal relationship.

Because the analysis required a documented quantitative EF, the findings pertain to patients in whom such a measurement was available. The available study records did not provide a validated screening denominator before application of the quantitative EF requirement or the numbers of otherwise eligible patients not enrolled because consent could not be obtained. The participating centres may not represent all regions of India. NYHA class, hospitalization, mortality, quality of life, and changes in treatment over time were not available, and clinical outcomes could therefore not be compared between HF phenotypes.

Atrial fibrillation was not recorded as a distinct entity in the case report form, limiting interpretation of its relationship with HFpEF, stroke, and treatment patterns. Although quantitative EF provided a consistent basis for phenotype classification, EF was available at a single time point only, so previous changes in EF could not be reconstructed. Although HF phenotypes were classified consistently using quantitative EF thresholds, HFpEF was not independently re-adjudicated using a uniform study-specific diagnostic algorithm incorporating structural, functional, and biomarker criteria. Some heterogeneity within the HFpEF group therefore cannot be excluded. Potential causes of heart failure were clinician-recorded, were not centrally adjudicated, and were not mutually exclusive. Whether enrolment was consecutive could not be verified from the available study records, and some selection bias therefore cannot be excluded.

## Conclusion

In this large multicentre outpatient cohort from India, HFpEF was characterized by a predominantly hypertensive, metabolic, and vascular comorbidity profile, including a higher prevalence of stroke. HFrEF was more strongly associated with ischaemic heart disease and higher NT-proBNP concentrations. Higher BMI and systolic blood pressure were independently associated with higher odds of HFpEF, while higher NT-proBNP concentrations and diabetes were associated with lower odds of HFpEF. These findings support phenotype-specific approaches to outpatient heart failure care and highlight the clinical relevance of natriuretic peptide assessment and blood pressure management. Given the cross-sectional design, these associations should be considered hypothesis-generating. Prospective studies with longitudinal EF measurements and clinical outcomes are needed to assess their temporal and prognostic significance.

## Supplementary Material

xvag236_Supplementary_Data

## Declarations

### Data availability

The data that support the findings of this study are available from the corresponding author upon reasonable request and with appropriate ethical approval.

### Pre-registered Clinical Trial Number

None supplied.

## References

[1] Savarese G, Becher PM, Lund LH, Seferovic P, Rosano GMC, Coats AJS. Global burden of heart failure: a comprehensive and updated review of epidemiology. Cardiovasc Res 2023;118:3272–87. 10.1093/cvr/cvac013 35150240

[2] Huffman MD, Prabhakaran D. Heart failure: epidemiology and prevention in India. Natl Med J India 2010;23:283–8. 10.1111/j.1751-7141.2011.01011 PMC3913650 21250584

[3] Harikrishnan S, Bahl A, Roy A, Mishra A, Prajapati J, Manjunath CN, et al Clinical profile and 90 day outcomes of 10 851 heart failure patients across India: National Heart Failure Registry. ESC Heart Fail 2022;9:3898–908. 10.1002/ehf2.14096 PMC9773752 36214477

[4] Ambrosy AP, Fonarow GC, Butler J, Chioncel O, Greene SJ, Vaduganathan M, et al The global health and economic burden of hospitalizations for heart failure: lessons learned from hospitalized heart failure registries. J Am Coll Cardiol 2014;63:1123–33. 10.1016/j.jacc.2013.11.053 24491689

[5] Samson R, Jaiswal A, Ennezat PV, Cassidy M, Le Jemtel TH. Clinical phenotypes in heart failure with preserved ejection fraction. J Am Heart Assoc 2016;5:e002477. 10.1161/JAHA.115.002477 PMC4859363 26811159

[6] Dunlay SM, Roger VL, Redfield MM. Epidemiology of heart failure with preserved ejection fraction. Nat Rev Cardiol 2017;14:591–602. 10.1038/nrcardio.2017.65 28492288

[7] Inamdar AA, Inamdar AC. Heart failure: diagnosis, management and utilization. J Clin Med 2016;5:62. 10.3390/jcm5070062 PMC4961993 27367736

[8] Metra M, Teerlink JR. Heart failure. Lancet 2017;390:1981–95. 10.1016/S0140-6736(17)31071-1 28460827

[9] Palazzuoli A, Evangelista I, Beltrami M, Pirrotta F, Tavera MC, Gennari L, et al Clinical, laboratory and lung ultrasound assessment of congestion in patients with acute heart failure. J Clin Med 2022;11:1642. 10.3390/jcm11061642 PMC8953698 35329969

[10] Heidenreich PA, Bozkurt B, Aguilar D, Allen LA, Byun JJ, Colvin MM, et al 2022 AHA/ACC/HFSA clinical practice guideline on heart failure. Circulation 2022;145:e895–1032. 10.1161/CIR.0000000000001063

[11] Authors/Task Force Members; McDonagh TA, Metra M, Adamo M, Gardner RS, Baumbach A, et al 2023 focused update of the 2021 ESC guidelines for the diagnosis and treatment of acute and chronic heart failure. Eur J Heart Fail 2024;26:5–17. 10.1002/ejhf.3024

[12] Kragelund C, Grønning B, Køber L, Hildebrandt P, Steffensen R. N-Terminal Pro–B-Type natriuretic peptide and long-term mortality in stable coronary heart disease. N Engl J Med 2005;352:666–75. 10.1056/NEJMoa042330 15716560

[13] Borlaug BA, Redfield MM. Diastolic and systolic heart failure are distinct phenotypes of the heart failure syndrome. Circulation 2011;123:2006–14. 10.1161/CIRCULATIONAHA.110.954388 PMC3420141 21555723

[14] Gevaert AB, Kataria R, Zannad F, Sauer AJ, Damman K, Sharma K, et al Heart failure with preserved ejection fraction: recent concepts in diagnosis, mechanisms and management. Heart 2022;108:1342–50. 10.1136/heartjnl-2021-319605 35022210

[15] Tan C, Dinh D, Brennan A, Hare DL, Kaye D, Lefkovits J, et al Characteristics and clinical outcomes in patients with heart failure with preserved ejection fraction compared to heart failure with reduced ejection fraction: insights from the VCOR heart failure snapshot. Heart Lung Circ 2022;31:623–8. 10.1016/j.hlc.2021.09.019 34742643

[16] Ponikowski P, Anker SD, AlHabib KF, Cowie MR, Force TL, Hu S, et al Heart failure: preventing disease and death worldwide. ESC Heart Fail 2014;1:4–25. 10.1002/ehf2.12005 28834669

[17] Harikrishnan S, Sanjay G, Anees T, Viswanathan S, Vijayaraghavan G, Bahuleyan CG, et al Clinical presentation, management, in-hospital and 90-day outcomes of heart failure patients in Trivandrum, Kerala, India: the Trivandrum heart failure registry. Eur J Heart Fail 2015;17:794–800. 10.1002/ejhf.283 26011246

[18] Heidenreich PA, Bozkurt B, Aguilar D, Allen LA, Byun JJ, Colvin MM, et al 2022 AHA/ACC/HFSA guideline for the management of heart failure. JACC 2022;79:e263–421. 10.1016/j.jacc.2021.12.012 35378257

[19] Lund LH, Crespo-Leiro MG, Laroche C, Zaliaduonyte D, Saad AM, Fonseca C, et al Heart failure in Europe: guideline-directed medical therapy use and decision making in chronic and acute, pre-existing and de novo, heart failure with reduced, mildly reduced, and preserved ejection fraction—the ESC EORP heart failure III registry. Eur J Heart Fail 2024;26:2487–501. 10.1002/ejhf.3445 PMC11683873 39257278

[20] Shukkoor AA, George NE, Radhakrishnan S, Velusamy S, Gopalan R, Kaliappan T, et al Clinical characteristics and outcomes of patients admitted with acute heart failure: insights from a single-center heart failure registry in South India. Egypt Heart J 2021;73:38. 10.1186/s43044-021-00161-w PMC8088418 33932180

[21] Paulus WJ, Tschöpe C. A novel paradigm for heart failure with preserved ejection fraction: comorbidities drive myocardial dysfunction and remodeling through coronary microvascular endothelial inflammation. J Am Coll Cardiol 2013;62:263–71. 10.1016/j.jacc.2013.02.092 23684677

[22] Kozman K, Ferrannini G, Benson L, Dahlström U, Hage C, Savarese G, et al Etiology of heart failure across the ejection fraction Spectrum and association with prognosis. JACC Heart Fail 2025;13:102491. 10.1016/j.jchf.2025.03.037 40527152

[23] Vaduganathan M, Fonarow GC, Greene SJ, DeVore AD, Kavati A, Sikirica S, et al Contemporary treatment patterns and clinical outcomes of comorbid diabetes Mellitus and HFrEF: the CHAMP-HF registry. JACC Heart Fail 2020;8:469–80. 10.1016/j.jchf.2019.12.015 32387066

[24] Genitsaridi I, Salpea P, Salim A, Sajjadi SF, Tomic D, James S, et al 11th edition of the IDF diabetes atlas: global, regional, and national diabetes prevalence estimates for 2024 and projections for 2050. Lancet Diabetes Endocrinol 2026;14:149–56. 10.1016/S2213-8587(25)00299-2 41412135

[25] Jia G, Hill MA, Sowers JR. Diabetic cardiomyopathy: an update of mechanisms contributing to this clinical entity. Circ Res 2018;122:624–38. 10.1161/CIRCRESAHA.117.311586 PMC5819359 29449364

[26] Lehrke M, Marx N. Diabetes Mellitus and heart failure. Am J Med 2017;130:S40–50. 10.1016/j.amjmed.2017.04.010 28526183

[27] Sakamoto D, Sotomi Y, Matsuoka Y, Nakatani D, Okada K, Sunaga A, et al Prognostic utility and cutoff differences in NT-proBNP levels across subgroups in heart failure with preserved ejection fraction: insights from the PURSUIT-HFpEF registry. J Card Fail 2025;31:771–80. 10.1016/j.cardfail.2024.10.440 39557334

[28] Januzzi JL, Butler J, Fombu E, Maisel A, McCague K, Piña IL, et al Rationale and methods of the prospective study of biomarkers, symptom improvement, and ventricular remodeling during sacubitril/valsartan therapy for heart failure (PROVE-HF). Am Heart J 2018;199:130–6. 10.1016/j.ahj.2017.12.021 29754651

[29] Khan SS, Beach LB, Yancy CW. Sex-based differences in heart failure. JACC 2022;79:1530–41. 10.1016/j.jacc.2022.02.013 35422249

[30] Hansen JL, Tarkia M, Vasilevska M, Sandin P, Asllanaj E, Wammes K, et al Trends in healthcare costs in heart failure and its clinical phenotypes during the implementation of SGLT2 inhibitors: a Finnish registry study. Clinicoecon Outcomes Res 2026;18:1–11. 10.2147/CEOR.S576426 PMC12990818 41847572

[31] McDonagh TA, Metra M, Adamo M, Gardner RS, Baumbach A, Böhm M, et al 2021 ESC guidelines for the diagnosis and treatment of acute and chronic heart failure. Eur Heart J 2021;42:3599–726. 10.1093/eurheartj/ehab368 34922348

[32] Armstrong PW, Pieske B, Anstrom KJ, Ezekowitz J, Hernandez AF, Butler J, et al Vericiguat in patients with heart failure and reduced ejection fraction. N Engl J Med 2020;382:1883–93. 10.1056/NEJMoa1915928 32222134

[33] Armstrong PW, Lam CSP, Anstrom KJ, Ezekowitz J, Hernandez AF, O’Connor CM, et al Effect of vericiguat vs placebo on quality of life in patients with heart failure and preserved ejection fraction: the VITALITY-HFpEF randomized clinical trial. JAMA 2020;324:1512–21. 10.1001/jama.2020.15922 PMC7576403 33079152

[34] Dorairaj P, Uthirapathi I, B K, Janakiraman E, Thiruvenkadam P, Mohammed SBK, et al Practical Guideline-Directed Medical Therapy (PGDMT) in heart failure with reduced and mildly reduced ejection fraction—real world multicenter cross-sectional interim analysis at enrollment: data from Associates for Cardiology Education and Research Chronic Heart Failure (ACERT CHEF) registry. J Card Fail 2026:S1071-9164(26)00183-1. 10.1016/j.cardfail.2026.03.021 41912005

[35] Anker SD, Butler J, Filippatos G, Ferreira JP, Bocchi E, Böhm M, et al Empagliflozin in heart failure with a preserved ejection fraction. N Engl J Med 2021;385:1451–61. 10.1056/NEJMoa2107038 34449189

[36] Solomon SD, McMurray JJV, Claggett B, de Boer RA, DeMets D, Hernandez AF, et al Dapagliflozin in heart failure with mildly reduced or preserved ejection fraction. N Engl J Med 2022;387:1089–98. 10.1056/NEJMoa2206286 36027570

[37] Pitt B, Pfeffer MA, Assmann SF, Boineau R, Anand IS, Claggett B, et al Spironolactone for heart failure with preserved ejection fraction. N Engl J Med 2014;370:1383–92. 10.1056/NEJMoa1313731 24716680

[38] Solomon SD, McMurray JJV, Vaduganathan M, Claggett B, Jhund PS, Desai AS, et al Finerenone in heart failure with mildly reduced or preserved ejection fraction. N Engl J Med 2024;391:1475–85. 10.1056/NEJMoa2407107 39225278

[39] Solomon SD, McMurray JJV, Anand IS, Ge J, Lam CSP, Maggioni AP, et al Angiotensin-neprilysin inhibition in heart failure with preserved ejection fraction. N Engl J Med 2019;381:1609–20. 10.1056/NEJMoa1908655 31475794

[40] Kittleson MM, Panjrath GS, Amancherla K, Davis LL, Deswal A, Dixon DL, et al 2023 ACC expert consensus decision pathway on management of heart failure with preserved ejection fraction: a report of the American College of Cardiology solution set oversight committee. J Am Coll Cardiol 2023;81:1835–78. 10.1016/j.jacc.2023.03.393 37137593

[41] Kosiborod MN, Abildstrøm SZ, Borlaug BA, Butler J, Rasmussen S, Davies M, et al Semaglutide in patients with heart failure with preserved ejection fraction and obesity. N Engl J Med 2023;389:1069–84. 10.1056/NEJMoa2306963 37622681

[42] Packer M, Zile MR, Kramer CM, Baum SJ, Litwin SE, Menon V, et al Tirzepatide for heart failure with preserved ejection fraction and obesity. N Engl J Med 2025;392:427–37. 10.1056/NEJMoa2410027 39555826

