# Supplementary material for: Demographics and clinical characteristics of Indian patients with heart failure visiting outpatient clinics: a cross-sectional observational study
Source: ESC Heart Fail. 2026 Sep 10;13(5):xvag236. doi: 10.1093/eschf/xvag236 (PMC13639485; doi:10.1093/eschf/xvag236)
Supplement: Supplementary file 1 [file xvag236_supplementary_data.docx]

**Table S1. Characteristics of patients included in the analytical cohort and patients excluded because of an out-of-range quantitative EF.**

| **Characteristic** | **Included in analytical cohort, N = 1,207** | **Excluded: EF outside plausible range, N = 43** | **Absolute SMD** |
| --- | --- | --- | --- |
| **Age, years** | **61.7 (9.6) [n = 1,207]** | **63.2 (11.1) [n = 43]** | **0.15** |
| **Male sex** | **881 (73.0%)** | **31 (72.1%)** | **0.02** |
| **Height, cm** | **163.4 (9.9) [n = 1,207]** | **163.0 (15.2) [n = 43]** | **0.04** |
| **Weight, kg** | **71.1 (11.2) [n = 1,207]** | **77.1 (9.8) [n = 43]** | **0.57** |
| **Body mass index, kg/m²** | **26.8 (4.6) [n = 1,204]** | **29.8 (7.2) [n = 43]** | **0.50** |
| **Systolic blood pressure, mmHg** | **130.0 (28.3) [n = 1,192]** | **151.7 (35.4) [n = 42]** | **0.68** |
| **Diastolic blood pressure, mmHg** | **84.7 (16.1) [n = 1,193]** | **105.9 (26.7) [n = 43]** | **0.96** |
| **Heart rate, bpm** | **85.5 (14.3) [n = 1,191]** | **87.1 (21.7) [n = 43]** | **0.09** |
| **Values are mean (SD) [n = number with a documented value] or n (%).** | | | |
| **SMD = standardized mean difference. Absolute SMDs are reported to describe the magnitude of differences between patients included in the analytical cohort and patients excluded because their documented quantitative EF was outside the prespecified plausible range.** | | | |
| **The comparison is descriptive. Because only 43 patients were excluded, it is not intended to demonstrate equivalence between the two groups, and no p values are reported.** | | | |

**Figure S1.** Patient flow for the EF-phenotyping analytical cohort.
